# Densovirus Is a Mutualistic Symbiont of a Global Crop Pest (*Helicoverpa armigera*) and Protects against a Baculovirus and Bt Biopesticide

**DOI:** 10.1371/journal.ppat.1004490

**Published:** 2014-10-30

**Authors:** Pengjun Xu, Yongqiang Liu, Robert I. Graham, Kenneth Wilson, Kongming Wu

**Affiliations:** 1 State Key Laboratory for Biology of Plant Diseases and Insect Pests, Institute of Plant Protection, Chinese Academy of Agricultural Sciences, Beijing, People's Republic of China; 2 Tobacco Research Institute, Chinese Academy of Agricultural Sciences, Qingdao, People's Republic of China; 3 Lancaster Environment Centre, Lancaster University, Lancaster, United Kingdom; University of Cambridge, United Kingdom

## Abstract

Mutualistic associations between symbiotic bacteria and their hosts are common within insect systems. However, viruses are often considered as pathogens even though some have been reported to be beneficial to their hosts. Herein, we report a novel densovirus, *Helicoverpa armigera* densovirus-1 (HaDNV-1) that appears to be beneficial to its host. HaDNV-1 was found to be widespread in wild populations of *H. armigera* adults (>67% prevalence between 2008 and 2012). In wild larval populations, there was a clear negative interaction between HaDNV-1 and *H. armigera* nucleopolyhedrovirus (HaNPV), a baculovirus that is widely used as a biopesticide. Laboratory bioassays revealed that larvae hosting HaDNV-1 had significantly enhanced resistance to HaNPV (and lower viral loads), and that resistance to *Bacillus thuringiensis* (Bt) toxin was also higher at low doses. Laboratory assays indicated that the virus was mainly distributed in the fat body, and could be both horizontally- and vertically-transmitted, though the former occurred only at large challenge doses. Densovirus-positive individuals developed more quickly and had higher fecundity than uninfected insects. We found no evidence for a negative effect of HaDNV-1 infection on *H. armigera* fitness-related traits, strongly suggesting a mutualistic interaction between the cotton bollworm and its densovirus.

## Author Summary

The old world cotton bollworm, *Helicoverpa armigera*, is one of the most significant pests of crops throughout Asia, Europe, Africa and Australia. Herein, we report a novel densovirus (HaDNV-1) which was widely distributed in wild populations of *H. armigera* and was beneficial to its host by increasing larval and pupal development rates, female lifespan and fecundity, suggesting a mutualistic interaction between the cotton bollworm and HaDNV-1. The cotton bollworm is currently widely controlled by the biopesticides *Bacillus thuringiensis* (Bt) toxin and the baculovirus HaNPV. It is therefore important to estimate the risk that the symbiotic virus will negatively impact on the efficiency of these biopesticides. Field and laboratory results suggest that HaDNV-1 infection significantly increases larval resistance to HaNPV and Bt toxin. These results have important implications for the selection of biopesticides for this species, and highlight the need for greater research into the elegant microbial interactions that may impact host individual and population dynamics.

## Introduction

The interactions between symbiotic species and their hosts are becoming increasingly understood within insect systems [1], [2], [3]. Symbionts form diverse evolutionary relationships that influence the life history of their host, from mutualistic, by protecting them from natural enemies or increasing their host's fitness though a variety of means [1], [4], [5], [6], [7], [8], to parasitic, either by decreasing their resistance to harmful microorganisms or their tolerance to environmentally harmful factors, or by killing them directly [9], [10], [11]. There is a growing literature on the mutualistic interactions between intracellular bacterial symbionts, such as *Wolbachia* and their insect hosts, in which the symbionts spread through the host population by increasing the fitness of infected hosts [1], [6], [12], [13]. However, viral mutualistic symbioses have rarely been reported. This may be because, as obligate symbionts, viruses have long been considered harmful to their host and are usually isolated from cadavers killed by the virus. Moreover, until relatively recently, laboratory techniques only had the capacity to shed light on overtly pathogenic viruses, and not covert beneficial ones [14], [15], [16]. The development of molecular and sequencing technology facilitates the discovery and analysis of non-pathogenic virus species, using techniques such as suppression subtractive hybridization (SSH) and RNA-seq [17], [18]. Generally, viruses isolated from healthy individuals may be conditionally beneficial to their hosts. Recently, these ‘good viruses’ have attracted more attention, largely due to the prospect of using them in applications such as gene therapy and as tools for gene manipulation [2], [19]. As defined by Roossinck, there are few examples of viral mutualistic symbioses in insects (identified as conveying benefit to the host without any detectable fitness costs) [2].

The cotton bollworm moth, *Helicoverpa armigera*, is a major migratory pest of cotton and other economically-important crops throughout Asia, Africa, Europe and Australasia [20], [21], [22]. In China, the introduction of Bt-cotton in the 1990s has seen a dramatic decline in the *H. armigera* moth population. However, there are signs of Bt-resistance emerging [23], [24], fueling renewed interest in other forms of biological pest control, including the use of host-specific viral pesticides, derived from densoviruses [25], small RNA viruses [26] and baculoviruses [27], [28], [29], [30], [31]. Previously, we reported a novel densovirus (HaDNV-1, from the family Parvoviridae) in *H. armigera* moths that possesses a monosense genome that is 4926 nucleotides in length and clustered with the members of the genus *Iteravirus* in phylogenetic analysis [32]. This has allowed further investigation into the interactions between HaDNV-1 and its host *H. armigera*, which we report here. The main objective of this study was to establish the ecological significance of this virus within the migratory *H. armigera* system. Specifically, we undertook experiments to determine the transmission strategies of HaDNV-1, the impact of HaDNV-1 infection on host fitness, including its capacity to modulate resistance to potentially lethal biopesticides, and the prevalence of HaDNV-1 in field populations of *H. armigera*. Our results show that HaDNV-1 can be both horizontally- and vertically-transmitted in *H. armigera*; that HaDNV-1 infection increases host-fitness by increasing larval/pupal development rate, female lifespan and egg/offspring production; and that it also enhances larval resistance to *H. armigera* nucleopolyhedrovirus (HaNPV), a widely-used biopesticide. Resistance to Bt Cry1Ac protoxin was also enhanced, but only at relatively low toxin concentrations. Overall, we found no evidence for a negative effect of densovirus infection on *H. armigera* fitness-related traits, strongly suggesting a mutualistic interaction between the cotton bollworm and HaDNV-1.

## Results

### Transmission strategies of HaDNV-1 and host-tissue distribution

To establish the modes of transmission of the densovirus HaDNV-1, we first produced an uninfected laboratory colony from a single breeding pair of *H. armigera* (NONINF strain). An infected strain (INF strain) was subsequently produced using neonate larvae from the NONINF strain, dosing them with either purified HaDNV-1 (10^8^/µl; method 1, see Materials and Methods) or filtered liquid from infected individuals (10^8^/µl; method 2, see Materials and Methods). Thus, our results indicated that HaDNV-1 could efficiently infect larvae by oral ingestion. The efficiency of infection with filtered liquid was higher than that of the purified virus (Table 1, Fig. S1A, S1B), suggesting that the purification process might have inactivated the virus in some way. We also found that individuals artificially infected with HaDNV-1 via peroral infection could efficiently transmit the viral infection to their offspring (Fig. S1C), and the same was true for naturally infected individuals (Fig. S1D), suggesting vertical transmission of the virus.

**Table 1:** The transmission mode of HaDNV-1.

Transmission mode	Individuals	Number testing +ve	Number testing −ve	Transmission efficiency (%)
Vertical	Female+/Male+	20	0	100
	Female−/Male+	14	4	78
	Female+/Male−	25	0	100
	Female−/Male−	0	14	0
Horizontal	Purified viruses	9	13	41
	Filtered liquid of infected adults	24	0	100

Infected individuals = “+”, uninfected individuals = “−”.

**Figure 1:**
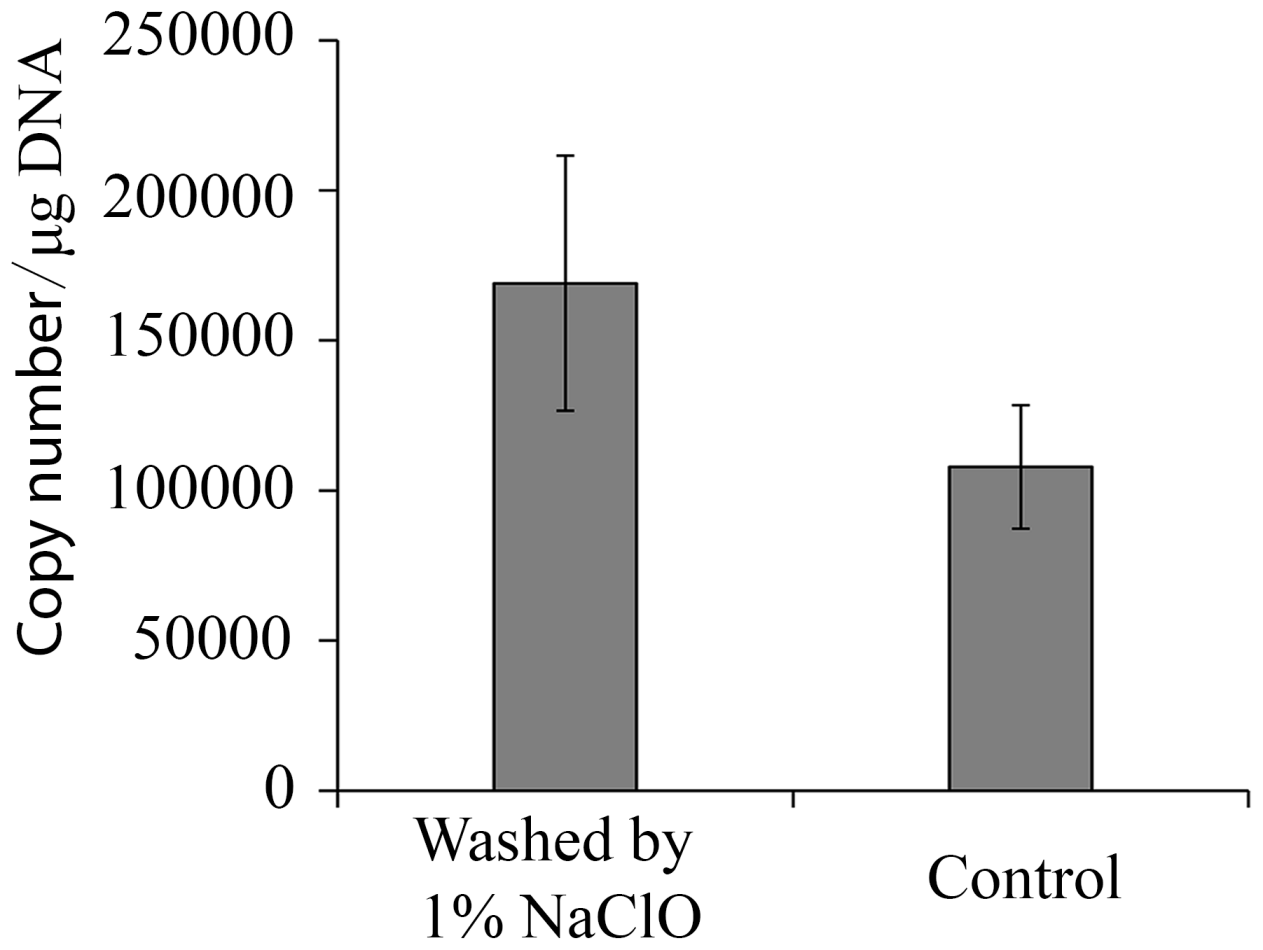
Viral load of HaDNV-1 in cotton bollworm eggs. Absolute quantification of HaDNV-1 copy number per µg of host DNA in eggs washed or non-washed in 1% sodium hypochlorite (n = 4).

HaDNV-1 was capable of being vertically-transmitted from both infected females and infected males, but transmission-efficiency was higher from infected females than males (Table 1, Fig. S1E, S1F, S1G). With qPCR, we tested whether vertical transmission of HaDNV-1 was due to virus contamination on the surface of the eggs (transovum), or whether the virus was transmitted within the egg itself (transovarial). HaDNV-1 titers were not significantly different between sodium hypochlorite-treated and non-treated eggs (t = 1.296, d.f. = 6, P = 0.24) (Fig. 1), suggesting that transovarial transmission was occurring.

To examine the possibility of horizontal transmission through ingestion of contaminated foodplant (as would be a possibility in wild populations), we placed uninfected neonate larvae in diet cells that had previously housed infected insects (n = 8). Our results indicated that horizontal virus transmission did not occur in this manner, despite our previous experimental evidence that larvae could be orally infected. To examine this further, we used a range of HaDNV-1 concentrations to infect larvae and subsequently examined virus intensity in host frass (faeces). As expected, larval infection rate was positively related to the magnitude of the HaDNV-1 oral challenge, with low infection rates at doses less than 10^6^/µl (Table 2); but even for larvae challenged with large viral doses, their frass contained only very low levels of HaDNV-1, with only 3 out of 20 samples containing more than 1×10^5^/mg and none with more than 5×10^5^/mg. Therefore, while we cannot exclude the possibility that horizontal transmission of HaDNV-1 may occur via the oral-fecal route, the viral levels in frass were very low and may not be sufficient for oral infection.

**Table 2:** Detection of HaDNV-1 infecting larvae dosed at a range of concentrations.

Concentrations (copy number/µl)	Number testing +ve	Number testing −ve	Infection rate (%)
10^8^	14	0	100
10^7^	12	3	80
10^6^	4	11	27
10^5^	2	13	13
10^4^	2	12	14

Infected individuals = “+ve”, uninfected individuals = “−ve”.

**Figure 2:**
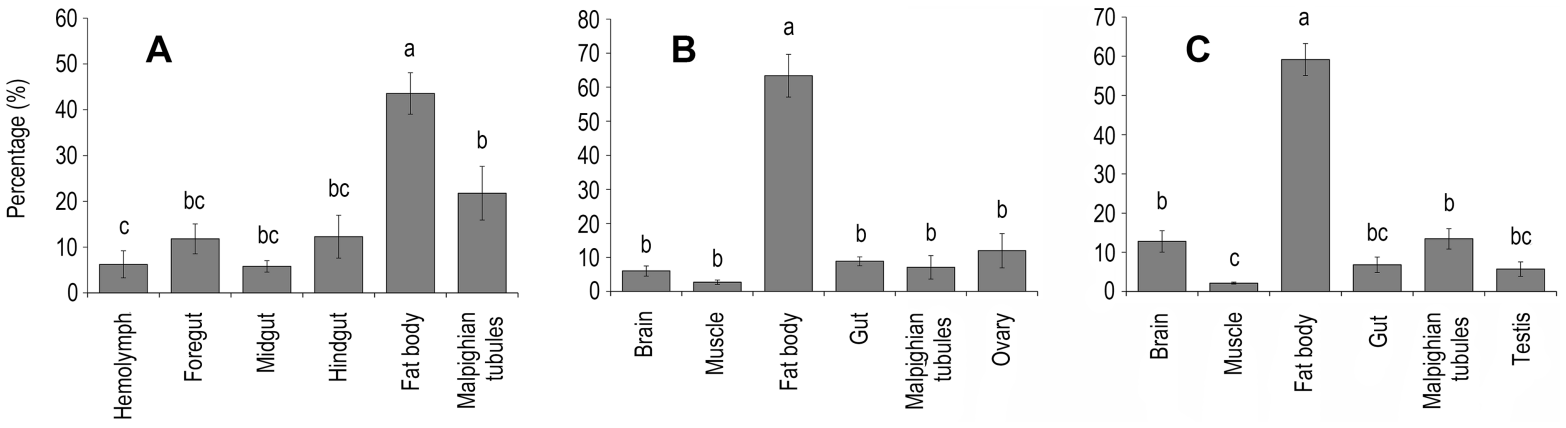
Tissue distribution of the HaDNV-1 in A) larvae, B) adult females and C) adult male cotton bollworms. Within each figure, significant differences ascribed using Tukey tests are shown using different letters. Percentage (%) = the ratio of HaDNV-1 in different tissues (per mg), as described in Methods 2.3 (larvae: n = 7; adult males: n = 6; adult females: n = 6). Means ± SE.

HaDNV-1 distribution was quantified within different host body tissues using qPCR. In both larvae and adults, HaDNV-1 titers were significantly higher in the fat body than in all other tissues: larvae: F = 11.098, d.f. = 5,36, P<0.0001 (Fig. 2A); adult females: F = 26.601, d.f. = 5,30, P<0.0001 (Fig. 2B); adult males: F = 44.560, d.f. = 5,30, P<0.0001 (Fig. 2C).

### Host range of HaDNV-1 infection

Using *H. armigera* as a control, we tested four other species of lepidopterans for their potential to act as alternative hosts for HaDNV-1, by attempting oral inoculation in *Spodoptera exigua*, *Spodoptera litura*, *Agrotis segetum* and *Agrotis ipsilon*. Results indicated that while oral inoculation with HaDNV-1 could successfully infect *H. armigera*, none of the four other species tested positive (Fig. S2A). We also tested field-captured adults of the closely-related species *H. assulta* but failed to find any HaDNV-1 positive individuals (n = 9; Fig. S2B). Based on these available data, it appears that infection with HaDNV-1 is host-specific to *H. armigera*.

### Effect of HaDNV-1 infection on the development, fecundity and adult longevity of *H. armigera*

**Figure 3:**
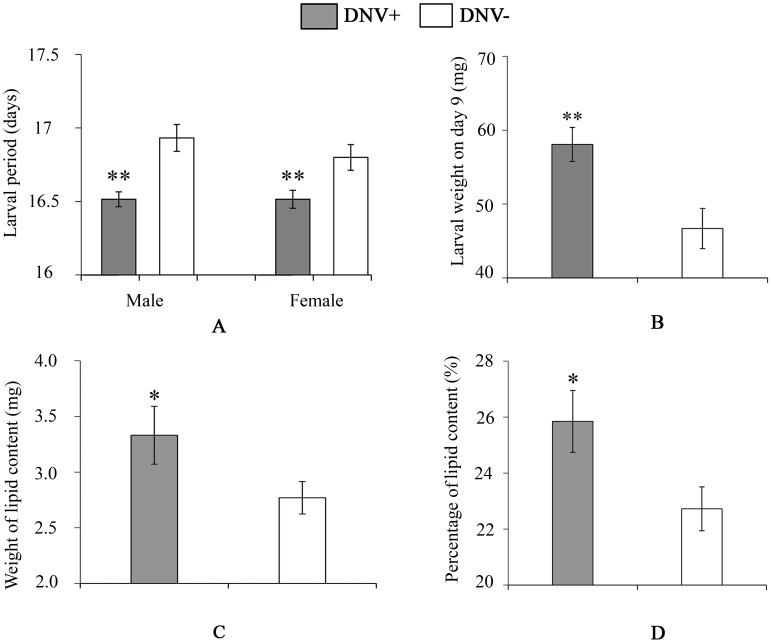
Larval life-history parameters of DNV- and DNV+ cotton bollworms. (A) Larval period (for male, n(DNV+) = 204, n(DNV−) = 177; for female, n(DNV+) = 169, n(DNV−) = 145). (B) Larval weight on day 9 (n = 19). (C) Lipid content in HaDNV-1 positive and negative larvae on day 9 (n(DNV+) = 19, n(DNV−) = 33). (D) Percentage of lipid content in HaDNV-1 positive and negative larvae on day 9 (n(DNV+) = 19, n(DNV−) = 33). DNV− = densovirus negative larvae, DNV+ = densovirus positive larvae. Means ± SE. * = P<0.05, ** = P<0.01.

**Figure 4:**
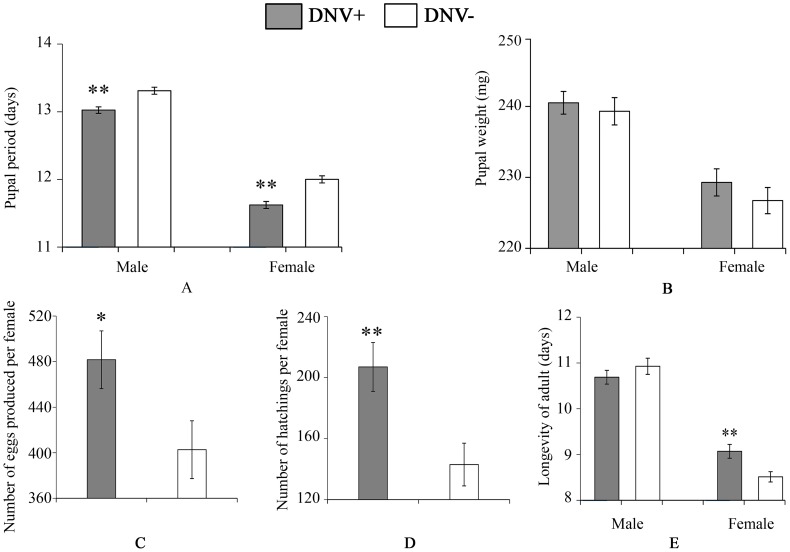
Pupal and adult life-history parameters of DNV− and DNV+ cotton bollworms. (A) Pupal period (for male, n(DNV+) = 204, n(DNV−) = 177; for female, n(DNV+) = 169, n(DNV−) = 145). (B) Pupal weight (for male, n(DNV+) = 204, n(DNV−) = 177; for female, n(DNV+) = 169, n(DNV−) = 145). (C) Number of eggs produced per female (n(DNV+) = 47, n(DNV−) = 48). (D) Egg hatch per female (n(DNV+) = 47, n(DNV−) = 48). (E) Longevity of adult (for male, n(DNV+) = 174, n(DNV−) = 195; for female, n(DNV+) = 174, n(DNV−) = 195). DNV− = densovirus negative larvae, DNV+ = densovirus positive larvae. Means ± SE. * = P<0.05, ** = P<0.01.

To quantify the impact of HaDNV-1 infection on *H. armigera* development, a number of bioassays were performed using neonate larvae orally inoculated with filtered liquid from either HaDNV-1 infected (DNV+) or non-infected (DNV−) individuals (Fig. S3A, S3B). Both male and female DNV+ individuals developed significantly more quickly than the control individuals in both the larval (female: t = 2.732, d.f. = 312, P = 0.0067, male: t = 4.147, d.f. = 379, P<0.001) (Fig. 3A) and pupal stages (female: t = 5.100, d.f. = 312, P<0.001, male: t = 4.057, d.f. = 379, P<0.001) (Fig. 4A). Between 7–11 days post-hatch (approximately 3^rd^–5^th^ instar) DNV+ larvae weighed significantly more than DNV- larvae by an average of ∼20% (GLMM with larval identity as a random term and log10-transformed larval weight as the dependent variable: Age (days): F = 2386.8, d.f. = 1,127, P<0.0001; HaDNV-1 infection status (+ve or −ve): F = 27.25, d.f. = 1,36, P<0.0001) (Fig. 3B, Fig. S4). However, their growth rates over this period did not differ, suggesting that densovirus effects on larval growth rate occurred prior to day 7 post-hatch (GLMM: interaction between infection status and age: F = 0.01, d.f. = 1,126, P = 0.91) (Fig. S4). A chloroform-wash assay indicated that at 9 days old, DNV+ larvae contained more lipid than DNV− individuals, measured as either lipid mass (t = 2.045, d.f. = 50, P = 0.046) or as a percentage of the whole body (t = 2.342, d.f. = 50, P = 0.023) (Fig. 3C, 4D). Larval mortality of DNV+ was significantly lower than DNV− (Table 3). However, there was no significant difference in pupal weight between DNV+ and DNV− insects (GLM: densovirus infection status: F = 0.99, d.f. = 1,692, P = 0.329; Sex: F = 41.08, d.f. = 1,693, P<0.0001; interaction term: F = 0.064, d.f. = 1,691, P = 0.80; female: t = 0.96, d.f. = 312, P = 0.34, male: t = 0.481, d.f. = 379, P = 0.63) (Fig. 4B), or pupation rate or eclosion rate between HaDNV-1 positive and HaDNV-1 negative insects (Table 3).

**Table 3:** The influence of HaDNV-1 on survival rates of cotton bollworm.

Index	DNV+ (%)	DNV− (%)	χ^2^_1_	n†	P value
Larval mortality	5.05 (±2.40)	8.16 (±3.25)	0.286	38	0.0322 *
Pupation rate	96.95 (±2.42)	96.47 (±4.20)	0.047	38	0.4828
Eclosion rate	93.13 (±3.67)	92.92 (±2.14)	0.002	38	0.8818
Hatching rate	42.57 (±5.66)	36.66 (±18.68)	58.84	95	0.0321 *

DNV+ = densovirus-infected; DNV− = non-infected individuals. Larval mortality = proportion of larvae dying before pupation; pupation rate = proportion of surviving larvae that successfully pupated; eclosion rate = proportion of pupae that successfully eclosed; hatching rate = proportion of each female's eggs that hatched.

† For larval mortality, pupation rate and eclosion rate, n = number of batches of 24 larvae; for hatch rate, n = number of females laying eggs.

To determine the effect of HaDNV-1 infection on adult life-history traits, we used individuals from the non-infected (NONINF) and infected (INF) strains; and their infection status was confirmed by PCR (Fig. S3C, S3D). Infected INF strain moths produced significantly more eggs (t = 2.172, d.f. = 93, P = 0.032; Fig. 4C) and more neonates (t = 3.026, d.f. = 93, P = 0.0032; Fig. 4D) than individuals from the uninfected NONINF strain. Egg viability (hatch-rate) was significantly higher in the INF strain than in the NONINF strain (Table 3). The life-span of densovirus-infected females was significantly longer than that of females that were virus-free (χ^2^_1_ = 13.5, d.f. = 1, P = 0.0002; Fig. 4E), but the longevity of males was not significantly different between the two strains (χ^2^ = 1.64, d.f. = 1, P = 0.2; Fig. 4E).

### Covariation of densovirus and baculovirus within field populations

In larval field-collections, there was a non-random association between the two viruses (Chi-square test with Yates' correction: χ^2^ = 35.63, d.f. = 1, P<0.0001). Thus, there were relatively fewer larvae infected with both HaDNV-1 and HaNPV than would be expected by chance alone (14% *versus* 20%). When split by year, this effect was significant in 2012, when the overall HaNPV prevalence was 61% (χ^2^ = 19.75, d.f. = 1, P<0.0001; proportion infected with both viruses = 20% observed *versus* 26% expected), but not in 2013, when HaNPV prevalence was just 4% (χ^2^ = 0.82, d.f. = 1, P = 0.36; 2% observed *versus* 2% expected) (Table S1).

In adult field-collections, the prevalence of HaDNV-1 infection was uniformly high each year between 2008 and 2012 - 87%, 81%, 77%, 68% and 67%, respectively (Fig. S5). However, there was evidence for a significant decline in densovirus prevalence over the five years (GLMM with location as a random effect: χ^2^_1_ = 39.06, P<0.0001). Despite high levels of baculovirus being observed in the larval field populations, we failed to detect any HaNPV-positive individuals in a random selection adult moths collected from four geographically diverse sites (n = 361 samples).

### Interaction between densovirus and microbial biopesticides

**Figure 5:**
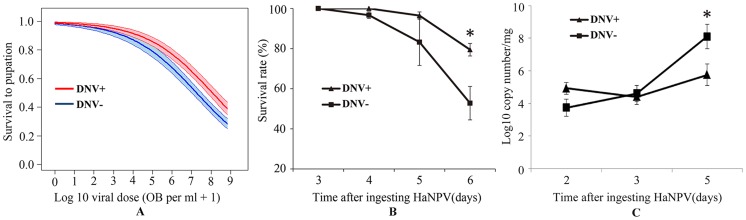
Relationship between the baculovirus HaNPV and the densovirus HaDNV-1 in cotton bollworm larvae. (A) Effect of HaNPV dose (log_10_-transformed number of occlusion bodies per ml) on larval survival to pupation. The thick lines are the fitted values and the shaded zones are the standard errors around these fitted values; blue line and shading = DNV− (control) larvae; red line and shading = DNV+ (densovirus-infected) larvae. The numbers of larvae that survived or died at differet concentrations (0, 10^6^, 10^7^, 10^8^, 10^9^ OB/ml) were 46/4, 38/8, 32/14, 20/32, 9/64 for DNV− individuals and 39/2, 31/10, 32/13, 31/17, 10/36 for DNV+ individuals. Temporal variation in (B) survival rate (%) (n = 216) and HaNPV copy numbers (log_10_-transformed) (C) (for day 2, n = 16; for day 3, n = 24; for day 5, n = 18) at different times after ingesting viruses. The concentrations of HaDNV-1 and HaNPV were 10^8^/µl and 10^8^ OBs/ml, respectively DNV− = densovirus negative larvae, DNV+ = densovirus positive larvae. Means ± SE. * = P<0.05, ** = P<0.01, based on t-tests at each time-point.

To determine the interaction between the densovirus HaDNV-1 and the baculovirus HaNPV, we first confirmed individuals from NONINF strain were NPV-free using PCR with specific primers. Then, NONINF strain neonates were inoculated with either HaDNV-1 (DNV+) or water (DNV− controls), and infections verified using PCR. Survival to pupation in larvae not exposed to HaNPV did not differ between DNV+ (95%) and DNV− (92%) larvae (χ^2^ = 0.27, d.f. = 1, P = 0.60). However, for those larvae exposed to the baculovirus, there was a significant difference between DNV+ and DNV− larvae in their susceptibility to HaNPV (GLM: HaDNV-1 infection-status: χ^2^ = 4.04, d.f. = 1, P = 0.044, parameter estimate ± standard error = 0.4645±0.2319), with densovirus-infected larvae suffering lower mortality rates for a given virus dose (GLM: log_10_ virus dose: χ^2^_1_ = 98.56, P<0.0001; LC50s = 3.13×10^7^ *versus* 9.10×10^7^ OB per ml, for DNV− and DNV+ larvae, respectively; Fig. 5A); the interaction between viral dose and infection status was marginally non-significant (dose*status: χ^2^ = 3.72, d.f. = 1, P = 0.054).

We tested the differences of HaNPV replication between HaDNV-1 positive and negative individuals by repeating the HaNPV bioassay with 10^8^ OBs/ml. The baculovirus bioassay indicated that there was no HaNPV-induced mortality in the control larvae that were exposed to water only, and that most mortality in the HaNPV-challenged larvae started at day 5 (120 h post-inoculation) (Fig. 5B). In NPV-challenged larvae, those carrying HaDNV-1 suffered significantly lower mortality overall than HaDNV-1 negative insects (Likelihood-ratio test: χ^2^ = 23.24, d.f. = 1, P<0.0001; linear coefficient (95% confidence interval) = 0.248 (0.134, 0.457)). Therefore, we collected samples before day 5 post-challenge to estimate HaNPV viral loads using qPCR. As would be expected, HaNPV titers (log-transformed) increased over time post-challenge and the rate of HaNPV titer increase was lower for HaDNV-1 positive larvae than in larvae lacking HaDNV-1, as indicated by a significant interaction term (linear model: Time post-challenge: F = 27.02, d.f. = 1,112, P<0.0001; DNV infection status: F = 5.69, d.f. = 1,112, P = 0.019; Time* DNV status interaction: F = 8.69, d.f. = 1,112, P = 0.0038; Fig. 5C). However, HaNPV titers were not directly correlated with HaDNV-1 titers in HaDNV-1 positive individuals (r = 0.066, n = 58, P = 0.623). These results suggest that HaDNV-1 protected *H. armigera* from HaNPV, possibly by slowing the accumulation of HaNPV.

**Figure 6:**
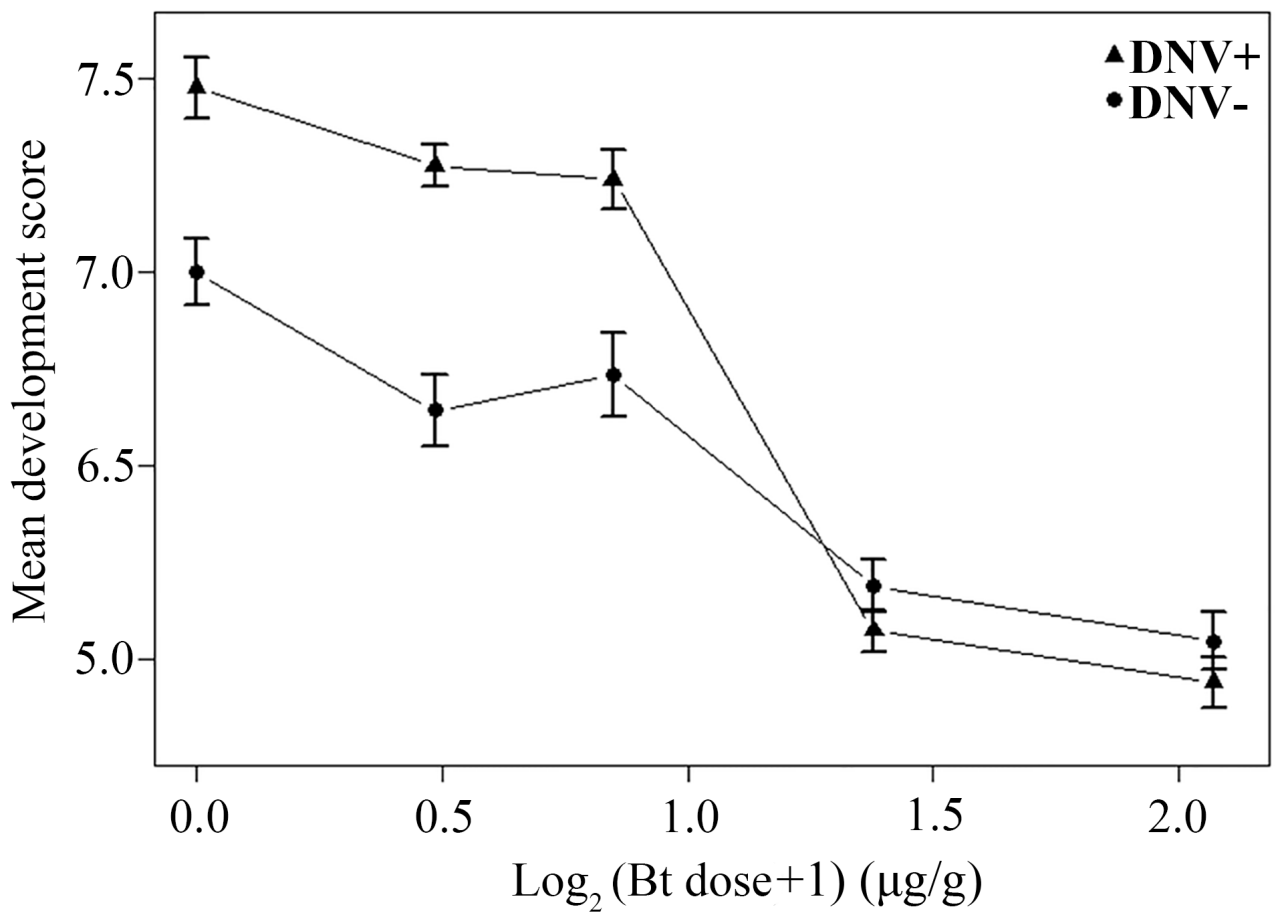
Relationship between dose of Bt toxin (log_2_-transformed) and mean development score for DNV+ and DNV- cotton bollworm larvae (averaged over days 4 to 9 post-challenge). Development score = a qualitative measure of average development stage achieved on a scale from 0 (death) to 11 (mid-4^th^ instar) (see Material and Methods for more details). DNV− = densovirus negative larvae, DNV+ = densovirus positive larvae.

A similar bioassay using the Bt toxin Cry1Ac instead of the baculovirus generated consistent results. As expected, larval development score increased over time and declined with increasing Bt dose (linear mixed-effects model with larval identity as a random term: Day: F = 18147.38, d.f. = 1,4172, P<0.0001; Log_2_Btdose: F = 1335.48, d.f. = 1,4172, P<0.0001). However, development was also influenced by the interaction between DNV infection status and the dose of Bt administered (DNV status: F = 120.21, d.f. = 1,4172, P<0.0001; DNV status * Bt dose interaction: F = 111.81, d.f. = 1,4172, P<0.0001), with the enhanced development of HaDNV-1 positive larvae at low Bt concentrations declining as Bt dose increased, such that mean development rate was independent of DNV infection status as Bt concentrations above 1.6 µg/g (Fig. 6). We also performed the bioassay with Bt cotton. As expected, there was a significant effect of Bt cotton on larval development rate, with development being significantly stunted in larvae exposed to the Bt plants (linear model: Diet: F = 63.74, d.f. = 1,476, P<0.001; mean score ± s.e.: Bt cotton = 1.717±0.153; non-Bt cotton = 3.529±0.167). However, whilst DNV positive larvae tended to have slightly higher development scores than DNV negative larvae (2.754±0.176 versus 2.492±0.164), this difference was non-significant and the interaction between DNV status and Bt exposure was also non-significant (DNV status: F = 1.336, d.f. = 1,476, P = 0.24; DNV status * Diet interaction: F = 0.0084, d.f. = 1,476, P = 0.93).

## Discussion

To date, viral mutualistic symbioses have attracted little attention and are rarely reported, most likely due to a lack of obvious pathogenicity within their insect hosts. In our study system, SSH was previously used to detect and isolate a novel densovirus (HaDNV-1) from healthy migratory cotton bollworms, *H. armigera* [32]. To date, most reported DNVs have been pathogenic to their hosts, even resulting in mortality, and as a result DNVs have been considered as potential biological control agents of insect pests [33], [34], [35], [36]. However, in our present study, for the first time, we show a mutualistic relationship without any detectable negative interactions between a DNV and its host.

Although endosymbionts of insects do have the capacity for horizontal transmission, they are usually transmitted via maternal inheritance [1], [3]. However, viral symbionts can be efficiently transmitted both vertically and horizontally [37], [38], [39], [40], [41], [42], [43], [44]. We found that HaDNV-1 was efficiently vertically-transmitted via both the paternal and maternal lines. This was most likely via transovarial infection, with the efficiency of transmission being higher from infected females than males. The results presented here also suggest that HaDNV-1 can be horizontally-transmitted to *H. armigera* by peroral infection of larvae, in a dose-dependent manner. However, we failed to detect horizontal transmission by diet contamination, suggesting that although larvae can be infected orally, peroral infection may only be possible at very high HaDNV-1 concentrations. Indeed, infection rate and intensity were both positively correlated with the magnitude of the HaDNV-1 challenge, and the frass of larvae contained only very low levels of HaDNV-1. This suggests that in the field, HaDNV-1 is likely to be almost exclusively transmitted vertically from parents to offspring. Previous studies suggest that DNVs may vary in their host ranges, for example *Junonia coenia* densovirus (JcDNV), *Mythimna loreyi* densovirus (MlDNV) and *Periplaneta fuliginosa* densovirus (PfDNV), all infect several host species, whereas *Galleria mellonella* densovirus (GmDNV) infects only one species [33]. Our results suggest that HaDNV-1 is also strongly host-specific following oral exposure, only infecting *H. armigera*.

Certain bacterial beneficial symbionts have been reported to benefit their hosts by shortening host development time and increasing host fecundity [1], [45]. However, evidence of viruses increasing host fecundity has rarely been reported. One exception is in a vector-virus complex in the whitefly *Bemisia tabaci*: a plant virus transmitted by *B. tabaci* was found to accelerate the population growth rate of its insect host [46]. In our system, HaDNV-1 infection intensity was greatest in the host fat body, suggesting that the virus might play a role in the development of *H. armigera*. Indeed, the significantly shortened development time and faster growth rate of *H. armigera* infected with HaDNV-1 could be mediated by the virus promoting the accumulation of fat body by the host. Our results showed that at 9 days old, HaDNV-1-infected larvae contained more lipid than uninfected larvae. The positive effect of the HaDNV-1 on these life-history traits, including egg/offspring production, suggests a possible mutualistic relationship. Taken together with the results of the baculovirus bioassay, these results suggest that HaDNV-1 benefits *H. armigera*, but is not an obligate microbe required by the host to survive.

The baculovirus HaNPV is a large double-stranded DNA virus, which was first isolated in China in 1975 and has since become an important biopesticide for a number of agricultural pests [27], [28], [29], [30], [31]. To determine the interaction between HaDNV-1 and HaNPV in *H. armigera*, we collected samples of larvae and adults from the field to determine the natural infection rates of HaDNV-1 and HaNPV. Most significantly, we found that there was a clear negative interaction between the two viruses across larval populations, with there being more insects infected with one or other of the viruses than would be expected by chance alone, and fewer with both viruses or neither. One possible explanation for this observation is that there is a negative interaction between the two viruses: perhaps HaDNV-1 increases susceptibility to HaNPV disease, resulting in those individuals with both viruses being more likely to die, as seen in larvae of the African armyworm moth, *Spodoptera exempta*, co-infected with *Wolbachia* and the baculovirus SpexNPV [9]. However, our results from the HaNPV-HaDNV-1 bioassay suggest the opposite, with HaDNV-1 infected larvae being significantly more resistant to HaNPV than those not carrying the densovirus. Therefore, it is likely that fewer than expected HaNPV-HaDNV-1 co-infected individuals were detected in field populations because HaDNV-1 protects its host against HaNPV infection. Our qPCR assay supported this hypothesis: HaNPV was found to accumulate in HaDNV-1 infected larvae at a slower rate than in uninfected larvae. Another possibility to explain the dearth of co-infected individuals is that rather than there being a direct interaction between the viruses, the interaction is indirect. Baculoviruses only infect the larval stages of Lepidoptera and early larval instars are generally more susceptible to viral infection (via oral ingestion) than older larvae, possibly because they slough virus-infected midgut cells at a slower rate [47]. If a larva can grow more quickly than its peers in the same cohort, then it will be less susceptible to virus infection and potentially “escape” disease (via this developmental resistance mechanism). Consistent with this, we found that HaDNV-1-positive larvae developed faster than HaDNV-1-negative larvae (Fig. 3) and accumulated HaNPV at a slower rate (Fig. 5C). In field populations of adults, the infection rate of HaDNV-1 remained high from 2008 to 2012 (more than 67%). However, we failed to detect any HaNPV baculovirus in any of the 361 adults sampled. Only the larval stage is susceptible to baculovirus infection and so one possible explanation for this is that most of the baculovirus-infected individuals are lost from the system before adulthood due to increased larval mortality, abnormal pupation, or unsuccessful eclosion [48]. Alternatively, enhanced resistance to HaNPV in the adult stage may effectively clear all viral infections gained in the larval stage.

Theory suggests that the presence of a beneficial symbiont should result in a high frequency of infection, spreading rapidly through a population until reaching infection fixation [1]. However, our data from adult moths suggest that although there was a high frequency of HaDNV-1 infection, there was also, perhaps unexpectedly, a steady decline in prevalence from 2008 to 2012, which would suggest an unidentified cost of DNV infection. One possible explanation for this decline is that the prevalence of HaDNV-1 is related to the recent widespread introduction to China of genetically-modified *Bacillus thuringiensis* (Bt) cotton [49], [50], [51]. For example, it might be that selection for Bt-resistance has selected against densovirus infection. If this was the case, then we might expect to observe a negative association between HaDNV-1 infection and resistance to Bt. However, in our laboratory experiment with Bt protoxin and artificial diet, HaDNV-1-positive larvae showed significantly higher resistance to Bt than HaDNV-1-negative larvae at low Bt concentrations (≤0.8 µg/g), while no significant difference was observed at high Bt concentrations (≥1.6 µg/g). Interestingly, the bioassay with Bt cotton plants showed that although HaDNV-1 positive larvae developed faster than negative ones, the difference was not statistically significant, possibly because the leaves of the Bt cotton used (at the seedling stage) contained a high concentration of Bt protein (about 1 µg/g) [52]. A related possibility is that densovirus prevalence is positively associated with the size of the *H. armigera* population in the wild, which has markedly declined since Bt-cotton was introduced [51], perhaps because horizontal transmission of the densovirus is enhanced at high population densities. The possibility of unknown competitive factors, including other microorganisms, can also not be excluded. Therefore, despite some evidence suggesting that HaDNV-1 could impact the population dynamics of *H. armigera*, our data are currently not comprehensive enough to explain the long-term dynamics of HaDNV-1, and more monitoring of field populations will be required to answer some of these intriguing questions.

### Conclusion

In conclusion, our studies to date suggest a mutualistic relationship between the cotton bollworm and HaDNV-1, in which the cotton bollworm appears to benefit from HaDNV-1 infection, with all host fitness parameters so far tested (larval growth rate, larval and pupal development rate, fertility, adult female lifespan, and resistance to baculovirus and low doses of Bt toxin) enhanced at no detectable cost. The study of beneficial viruses in both vertebrate and invertebrate systems has only relatively recently attracted researchers' attention [2], predominantly due to the explosion of new technologies that now make the detection of such organisms possible. It should be noted that the coevolution between viral mutualistic symbionts and their hosts could be an important factor to consider when studying the adaptability of insect host species. Illuminating the function of such viral symbionts may offer novel insights for future pest management strategies.

## Materials and Methods

### Insect culture and preparation of HaDNV-1 virus

Cotton bollworms (*H. armigera*) were reared using artificial diet [53] at 25±1°C with a 14:10, light:dark photoperiod. Adult moths were provided with 10% sugar and 2% vitamin complex. The colony was established from thirty breeding pairs captured at Langfang (Hebei province, China) in 2005. Individuals successfully producing offspring were tested for the presence of HaDNV-1, using the methods described below. Offspring from a single uninfected breeding pair were reared to produce the NONINF strain (uninfected) laboratory culture.

HaDNV-1 virus was isolated from migrating *H. armigera* adults captured in 2010 and 2011 using a vertical-pointing trap, and stored in liquid nitrogen [20]. Briefly, DNA was extracted from host tissues (except for the abdomens) of each individual, and PCR undertaken to detect the presence of HaDNV-1. Subsequently, the abdomens of positive individuals were divided into two groups: one group was used to purify the HaDNV-1 using the method described by La Fauce et al. (method 1) [54]; the other group was used to prepare a filtered liquid, containing an unpurified form of virus (method 2). Briefly, this second method involved grinding four abdomens under liquid nitrogen and transferring to 1 ml PBS buffer (0.01M, pH 7.4). The homogenate was centrifuged at 6500×g for 15 min at 4°C, and the liquid supernatant subsequently filtered with Sartorius Minisart 0.2 µm PES (Invitrogen, Grand Island, USA). The abdomens of negative individuals were filtered using the same method. Quantification of the viruses was performed using the qPCR method described below. All the samples were stored at −20°C.

### Virus detection and quantification

To detect the existence of HaDNV-1 in *H. armigera*, specific primers amplifying a 496 bp fragment, DVVPF/DVVPR (Table S2) were designed according to the genomic sequence of HaDNV-1. The PCR program was as follows: 30 s at 94°C, 30 s at 55°C, and 30 s at 72°C for 40 cycles. For detection of *H. armigera* nucleopolyhedrovirus (HaNPV), a pair of specific primers amplifying a fragment of 445 bp, NPVF/NPVR, were designed according to the open reading frame 14 (ORF14) of the genomic sequence of HaNPV. The PCR program was as follows: 30 s at 94°C, 30 s at 57°C, and 30 s at 72°C for 40 cycles.

For quantifying the copy numbers of HaDNV-1 and HaNPV, an absolute quantification qPCR methodology using a standard curve was performed [55]. Fragments containing the primers and probes of HaDNV-1 and HaNPV were amplified with our *de novo* primers (PF/PR for HaDNV-1, NPVF/NPVR for HaNPV) using the program: 30 s at 94°C, 30 s at 53°C, and 60 s at 72°C for 40 cycles, and cloned into the pEASY-T Cloning Vector (TransGen, Beijing, China). These plasmids were subsequently used for the quantification standard curve assay. qPCR was carried out with the TaqMan method in 20 µl reaction agent comprised of 1 µl of template DNA, 2×Premix Ex Taq (Takara, Japan), 0.2 µM of each primer and 0.4 µM probe, using a 7500 Fast Real-time PCR System (Applied Biosystems). Thermal cycling conditions were: 45 cycles of 95°C for 15 s, 60°C for 34 s. The DNA sample of each group was replicated three times. All primers used in this study were shown in Table S2. The equation of y = −1.052x+42.327 (y = the logarithm of plasmid copy number to base 2, x = Ct value, R^2^ = 0.9997) and y = −0.9861x+44.647 (y = the logarithm of plasmid copy number to base 2, x = Ct value, R^2^ = 0.9999) were used to calculate the copy number of HaDNV-1 and HaNPV, respectively.

### HaDNV-1 transmission and host tissue distribution

We constructed an infected line (INF strain) of *H. armigera* by orally infecting NONINF strain larvae with HaDNV-1 (from filtered liquid, method 2 - see above) and maintained them by vertical transmission of the virus, using the primers DVVPF/DVVPR to confirm successful establishment of HaDNV-1 infection. Subsequently, individuals from both NONINF strain and INF strain were used to determine the transmission modes of HaDNV-1. For vertical transmission, ♀+/♂−, ♀−/♂+, ♀+/♂+ and ♀−/♂− pairs were crossed and DNA from 3^rd^ instar offspring larvae used to probe for HaDNV-1.

For the diet contamination assay, (to determine horizontal transmission efficiency), infected individuals from the INF strain were reared in diet cells until the start of the 3^rd^ instar and then removed. Uninfected NONINF strain neonates were then placed in the vacated cells and reared to the pupal stage. DNA was extracted from the adults and probed for HaDNV-1 infection using PCR. Horizontal transmission of HaDNV-1 was determined using PCR with adult DNA as temples and different concentrations of the densovirus: 10^8^, 10^7^, 10^6^, 10^5^, 10^4^/µl. The frass of larvae from HaDNV-1 positive individuals were also quantified by qPCR, as described above.

To examine virus infection in different body tissues, DNA was extracted from body parts of infected individuals (both larval and adult stages) and the copy numbers of HaDNV-1 were quantified by qPCR. To account for individual variation, we first calculated the copy numbers per milligram of tissue and then summed all the copy numbers from different tissues from the same individual and the percentage of each tissue was statistically analyzed (larvae: n = 7; adult males: n = 6; adult females: n = 6).

### Quantification of HaDNV-1 in eggs

To further establish the role of vertical transmission in the life-cycle of the densovirus, we quantified HaDNV-1 infections in *H. armigera* eggs, primarily to distinguish between transovarial and transovum infection routes. Eggs from INF strain breeding pairs, which both of females and males were infected by HaDNV-1, were submerged in 1% sodium hypochlorite for 10 minutes. They were then filtered through a damp cloth, thoroughly rinsed, and allowed to dry. Four groups of hypochlorite-treated eggs (n = 50 eggs per group) were tested against non-treated eggs (control) and HaDNV-1 infections tested by qPCR.

### Quantifying HaDNV-1 impact on host development and fecundity

To test the impact of HaDNV-1 infection on the life table parameters of its host, neonate NONINF strain larvae were first orally inoculated with either filtered-liquid containing HaDNV-1, or filtered-liquid from uninfected individuals (control). One hundred NONINF strain neonates were placed in each treatment Petri-dish for 2 days to ensure that larvae ingested the treated diet. They were then transferred to a 24-well plate (one individual per well: diameter = 1.5 cm; height = 2 cm) until the 5^th^ larval instar; larvae were then individually reared in glass tubes until eclosion (diameter = 2 cm; height = 7.5 cm) (Fig. S6). The status of individuals was checked every day at 9:00 am. The weight of larvae from the 7^th^ to 11^th^ day post hatch, and the pupa on the 3^rd^ day were recorded. Fifth-instar larvae were randomly selected to estimate the infection rate of HaDNV-1 during the experiment. This bioassay was replicated twice (n = 288 and n = 168 individuals, respectively). Individuals dying within 24 hours of the experimental set up were considered handling deaths, and excluded from the analysis.

In addition, newly eclosed adults from both the HaDNV-1 negative NONINF strain and HaDNV-1 positive INF strain were used to determine longevity, egg production and hatch rate. Three pairs of adults were put in each plastic cup (diameter = 8.5 cm; height = 10 cm) (Fig. S6). The experimental replicates were 3×77 for NONINF strain and 3×60 for INF strain, respectively. We recorded the number of eggs and newly hatched larvae every day. After death, individuals were used to detect HaDNV-1 via PCR. Data from failed matings were excluded.

### Lipid quantification in HaDNV-1 positive and negative individuals

To quantify the impact of HaDNV-1 infection on host growth, we measured relative lipid mass within larvae of *H. armigera*. Larvae 9 days post-hatch were chosen to compare the lipid content between HaDNV-1 positive (n = 19) and HaDNV-1 negative (n = 33) individuals. The protocol was undertaken as Clissold et al. [56]. Briefly, the larval samples were freeze-dried, weighed, chloroform-extracted 3 times, dried again and weighed. The lipid mass was calculated by subtracting the post-chloroform-wash mass from the pre-chloroform-wash mass.

### Baculovirus and Bt bioassays

To assess the capacity of HaDNV-1 to act as a beneficial symbiont, we quantified the interaction between HaDNV-1 and the common baculovirus pathogen HaNPV, via a series of laboratory bioassay studies. As previously described, neonate larvae were first treated with HaDNV-1 filtered liquid (either from HaDNV-1 infected or HaDNV-1 negative individuals). Two-day old larvae were then transferred to a 24-well plate and maintained on diet until the 9^th^ day after hatching. Individuals weighing between 5–11 mg (early third-instar stage) were chosen for the HaNPV bioassay. Purified powder of HaNPV at a concentration of 5×10^11^ occlusion bodies (OBs) per g was generously provided by Dr. Qilian Qin in the Institute of Zoology, Chinese Academy of Science, Beijing, China. Larvae were orally dosed with 4 treatments of HaNPV (30 larvae per treatment at: 0 (control), 1×10^6^, 1×10^7^, 1×10^8^, and 1×10^9^ OBs/ml). Only larvae that ingested all the NPV within a 24 h period were used for the bioassay. Larvae were subsequently monitored daily for NPV mortality until pupation, and all viral deaths stored at −20°C. PCR with specific primers was used to test for NPV in dead larvae with non-obvious symptoms.

To assess HaNPV infection levels in HaDNV-1 positive and negative individuals, we performed a separate HaNPV bioassay with 10^8^ OBs/ml. There were 24 individuals in each replicate and three replicates per treatment. Only larvae that ingested all the NPV within a 24 h period were used for the bioassay. The absolute quantification qPCR methodology was used to quantifying the copy numbers of HaNPV as described above. Survival analysis was conducted using Cox's proportional hazards model.

For the *Bacillus thuringiensis* bioassays, various concentrations of the Bt Cry1Ac protoxin were added and thoroughly mixed with standard artificial diet to obtain the desired concentrations (0 (control), 0.4 µg/g, 0.8 µg/g, 1.6 µg/g and 3.2 µg/g). After mixing, the diet solidified and solid 1 mg pieces were placed into each well of a 24-well plate and two-day old larvae infected or uninfected by HaDNV-1 were then transferred to each well (Fig. S6). There were 24 individuals in each replicate and three replicates per treatment. We graded the larvae from day 4 to day 9 after hatching according to the development rate: death = 0, early first instar stage = 1, middle first instar stage = 2, last first instar stage = 3, early second instar stage = 4, middle second instar stage = 5, last second instar stage = 6, early third instar stage = 7, middle third instar stage = 8, last third instar stage = 9, early fourth instar stage = 10, middle fourth instar stage = 11 [57].

At seedling stage with 5 leaves, we chose the new cotton 33B with Cry1Ac (Monsanto Company, Bt cotton) using Shi Yuan 321 (Shijiazhuang Acadamy of Agricultural Sciences, NonBt cotton) as control to perform the bioassay. Two-day old larvae infected or uninfected by HaDNV-1 were transferred to a 24-well plate with Bt-cotton or NonBt-cotton. There were 40 individuals in each replicate and three replicates per treatment. We graded the larvae after 7 days according to the development rate.

### Detection of HaDNV-1 and HaNPV in wild populations of *H. armigera*

Samples of larvae were collected at 7 locations in 2012 (Jinan, Dezhou and Taian, Shandong province; Cangzhou, Heibei province; Tianmen and Qianjing, Hubei province; Maanshan, Anhui province) and 6 locations in 2013 (Luohe, Luoyang, Yuanyang and Nanyang, Henan province; Langfang and Cangzhou, Hebei province). The infection rate of HaDNV-1 and HaNPV was determined using the PCR method described as above.

Samples of adults were collected at fifteen locations from 2008 to 2012: A = Xinxiang, Henan province; B = Dezhou; C = Langfang; D = Yantai Shandong province; E = Yancheng, Jiangsu province; F = Handan, Shandong province; G = Changde, Hunan province; H = Tianmen, I = Qianjiang; J = Maanshan; K = Taian; L = Luohe; M = Weinan, Shanxi province; N = Shihezi, O = Kashi, Xinjiang province. We also randomly selected four places to detect HaNPV in the populations, including site 1 in 2010 (54 samples), site 2 in 2010 (103 samples), site 4 in 2012 (104 samples) and site 13 in 2011 (100 samples).

### Host range of HaDNV-1

Using the same oral inoculation method as previously described (section 2.5), we chose four species of Lepidoptera (*Spodoptera exigua*, *Spodoptera litura*, *Agrotis segetum*, *Agrotis ipsilon*) to determine the host range of HaDNV-1 infection. We also collected nine adults of *H. assulta* from field populations, and PCR was used to detect HaDNV-1 infection.

### Statistics

Statistical analyses were conducted using STATA v.9.0 and R v3.0.1 [58]. Student's t-test or ANOVA with Tukey were used to determine the level of significance in the relative levels of HaDNV-1. Egg hatch rates and larval/pupal mortality, pupation and eclosion rates were determined using generalized linear models (GLMs) with binomial errors. Analysis of the NPV and Bt bioassay data was also conducted using GLMs with binomial errors. A generalised linear mixed effects model (GLMM) with binomial errors was used to determine temporal variation in HaDNV-1 infection rates. A GLMM with Gaussian errors was used to quantify variation in larval growth rates with larval identity included as a random term. Development following exposure to Bt toxin in artificial diet was analyzed using linear mixed effects models using the *lme* function in R, with larval identity as a random term to account for the repeated measures data structure.

### Accession number

The GenBank accession number of genomic sequence of HaDNV-1 and HaNPV were HQ613271 and AF303045, respectively.

## Supporting Information

Figure S1 **The transmission mode of HaDNV-1.** (A) PCR detection of HaDNV-1 following peroral inoculation with the filtered liquid. (B) PCR detection following inoculation with purified viruses. (C) The detection of HaDNV-1 in offspring of individuals which were artificially perorally infected with the filtered liquid. (D) The detection of HaDNV-1 in offspring of individuals which were naturally infected with HaDNV-1 (captured in 2012 from Jinan, Shandong province). Vertical transmission of HaDNV-1: (E) ♀+/♂+; (F) ♀−/♂+; (G) ♀+/♂− and (H) ♀−/♂−. M = marker (2 kb, 1 kb, 0.75 kb, 0.5 kb, 0.25 kb and 0.1 kb, respectively), “+” = positive control, “−” = negative control.
(TIF)

Figure S2 **The detection of HaDNV-1 host spectrum.** (a) Detection by peroral infection. Samples in the left of “M” were HaDNV-1 positive. Samples in the right of “M” were control. “−” stands for negative control. Se = *Spodoptera exigua*, Sl = *S. litura*, As = *A. segetum*, Ay = *A. ypsilon*. Ha = *H. armigera*. (b) Detection using samles of wild-captured *H. assult*. M = marker (Figure 1b), “+” stands for positive control.
(TIF)

Figure S3 **The detection of HaDNV-1 in sample used in bioassay.** The detection of HaDNV-1 both (a) DNA and (b) RNA in 5^th^ instar larvae infected by HaDNV-1 via oral inoculation in the bioassay experiment. The detection of HaDNV-1 in adults from (c) NONINF-strain and (d) INF-strain used in the experiment of egg production. M = marker (Figure 1b), “+” = positive control, “−” = negative control.
(TIF)

Figure S4 **Larval weight (log10-transformed) from day 7 to 11 after hatching.** For day 7, n = 7; day 8, n = 19; day 9, n = 19; day 10, n = 19; day 11, n = 19. DNV− = densovirus negative larvae, DNV+ = densovirus positive larvae. Means ± SE. * = P<0.05, ** = P<0.01.
(TIF)

Figure S5 **Distribution of HaDNV-1 in** ***H. armigera*** **from different populations.** The black proportion of circles stands for infected individuals, and the gray stands for uninfected individuals. Different letters stand for different places for collecting samples. Infection rates were 87.1% in 2008 (n = 170), 81.2% in 2009 (n = 373), 76.8% in 2010 (n = 699), 68% in 2011 (n = 544) and 67% in 2012 (n = 370). Infected size/uninfected size in 2008: A = 30/1, B = 33/3, C = 43/7, D = 26/1, E = 16/10; in 2009: A = 38/2, B = 27/5, C = 18/7, D = 113/11, E = 39/11, F = 14/4, G = 8/16, H = 14/9, I = 15/2, K = 18/2; in 2010: A = 101/6, B = 98/5, C = 70/40, D = 113/72, E = 26/1, F = 18/0, G = 17/3, I = 22/7, J = 25/25, K = 27/3, L = 20/0; in 2011: A = 17/2, B = 8/2, C = 95/3, D = 46/51, E = 12/9, F = 16/4, G = 23/4, H = 8/5, I = 17/2, K = 18/0, L = 13/3, M = 63/37, N = 4/10, O = 30/42; in 2012: A = 16/2, B = 37/39, C = 127/20, D = 28/49, F = 19/1, N = 21/11.
(TIF)

Figure S6 **Tools used in the bioassay.**
(TIF)

Table S1 **The detection of DNV and NPV in larvae of** ***H. armigera*** **.** “+” = infected, “−” = non-infected. “DNV+ and NPV+” = insects co-infected with both viruses.
(DOC)

Table S2 **Primers used in this study.**
(DOC)
