# Supplementary figures and images for: Densovirus Is a Mutualistic Symbiont of a Global Crop Pest (Helicoverpa armigera) and Protects against a Baculovirus and Bt Biopesticide
Source: PLoS Pathog. 2014 Oct 30;10(10):e1004490. doi: 10.1371/journal.ppat.1004490 (PMC4214819; doi:10.1371/journal.ppat.1004490)

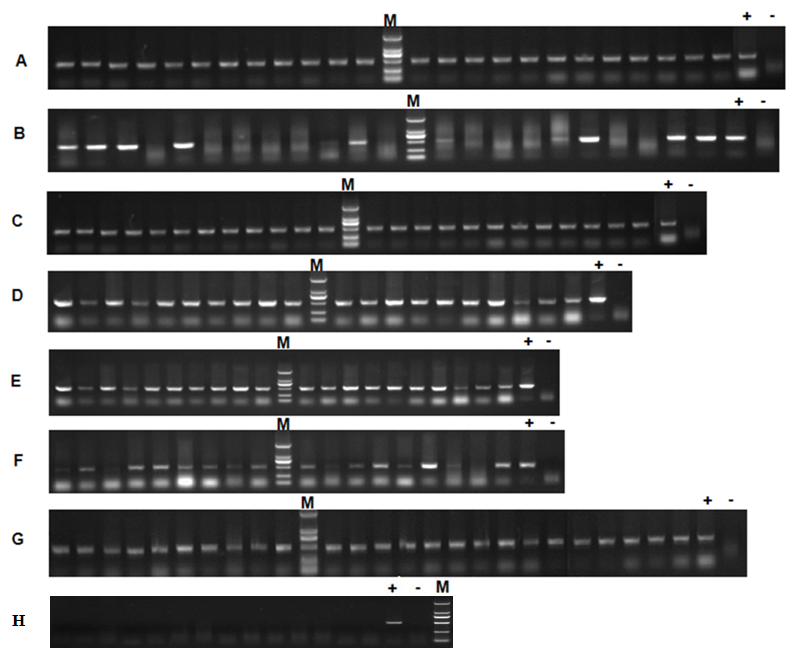

Supplement: Figure S1 — The transmission mode of HaDNV-1. (A) PCR detection of HaDNV-1 following peroral inoculation with the filtered liquid. (B) PCR detection following inoculation with purified viruses. (C) The detection of HaDNV-1 in offspring of individuals which were artificially perorally infected with the filtered liquid. (D) The detection of HaDNV-1 in offspring of individuals which were naturally infected with HaDNV-1 (captured in 2012 from Jinan, Shandong province). Vertical transmission of HaDNV-1: (E) ♀+/♂+; (F) ♀−/♂+; (G) ♀+/♂− and (H) ♀−/♂−. M = marker (2 kb, 1 kb, 0.75 kb, 0.5 kb, 0.25 kb and 0.1 kb, respectively), “+” = positive control, “−” = negative control. (TIF) [file ppat.1004490.s001.tif]

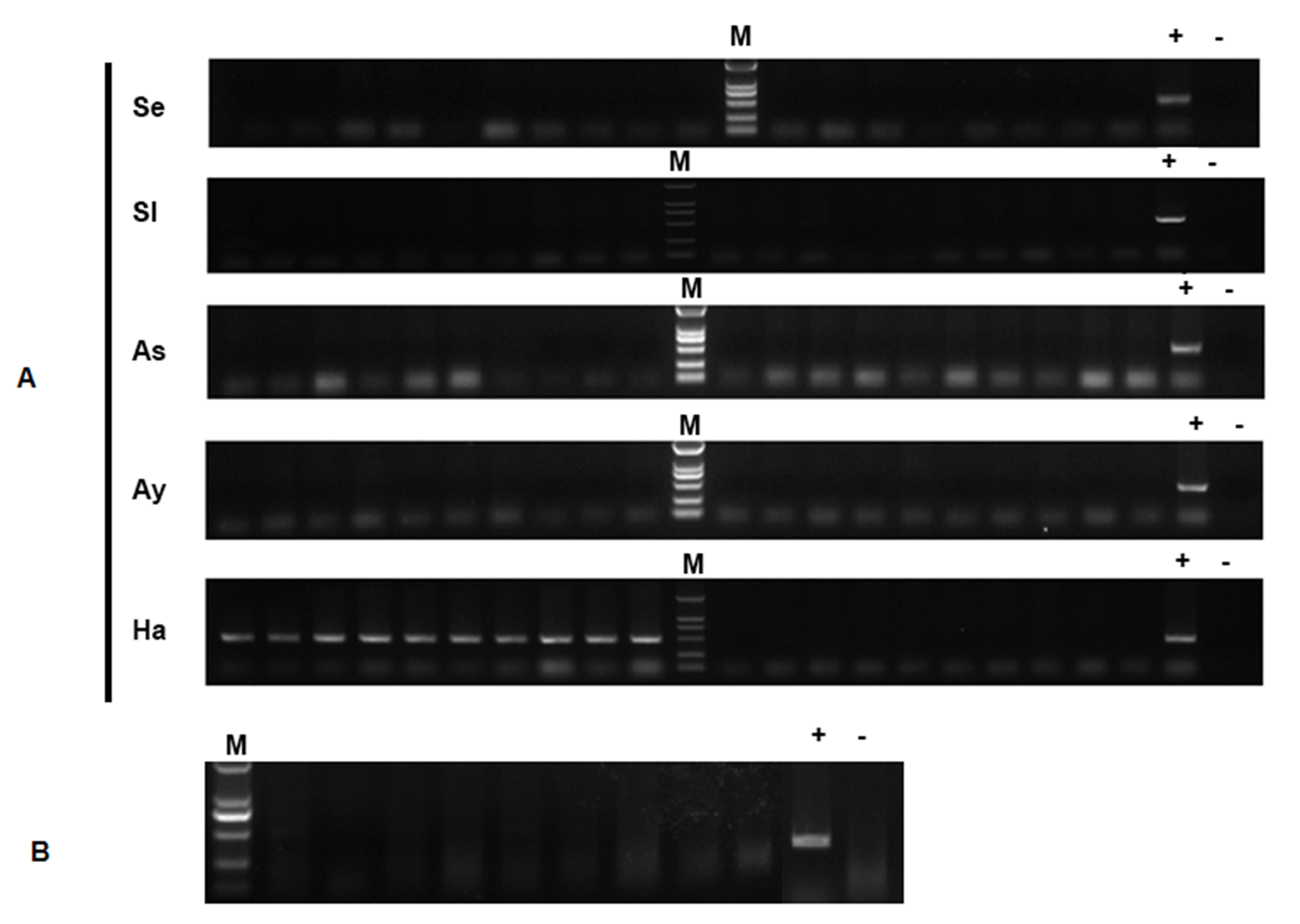

Supplement: Figure S2 — The detection of HaDNV-1 host spectrum. (a) Detection by peroral infection. Samples in the left of “M” were HaDNV-1 positive. Samples in the right of “M” were control. “−” stands for negative control. Se = Spodoptera exigua, Sl = S. litura, As = A. segetum, Ay = A. ypsilon. Ha = H. armigera. (b) Detection using samles of wild-captured H. assult. M = marker (Figure 1b), “+” stands for positive control. (TIF) [file ppat.1004490.s002.tif]

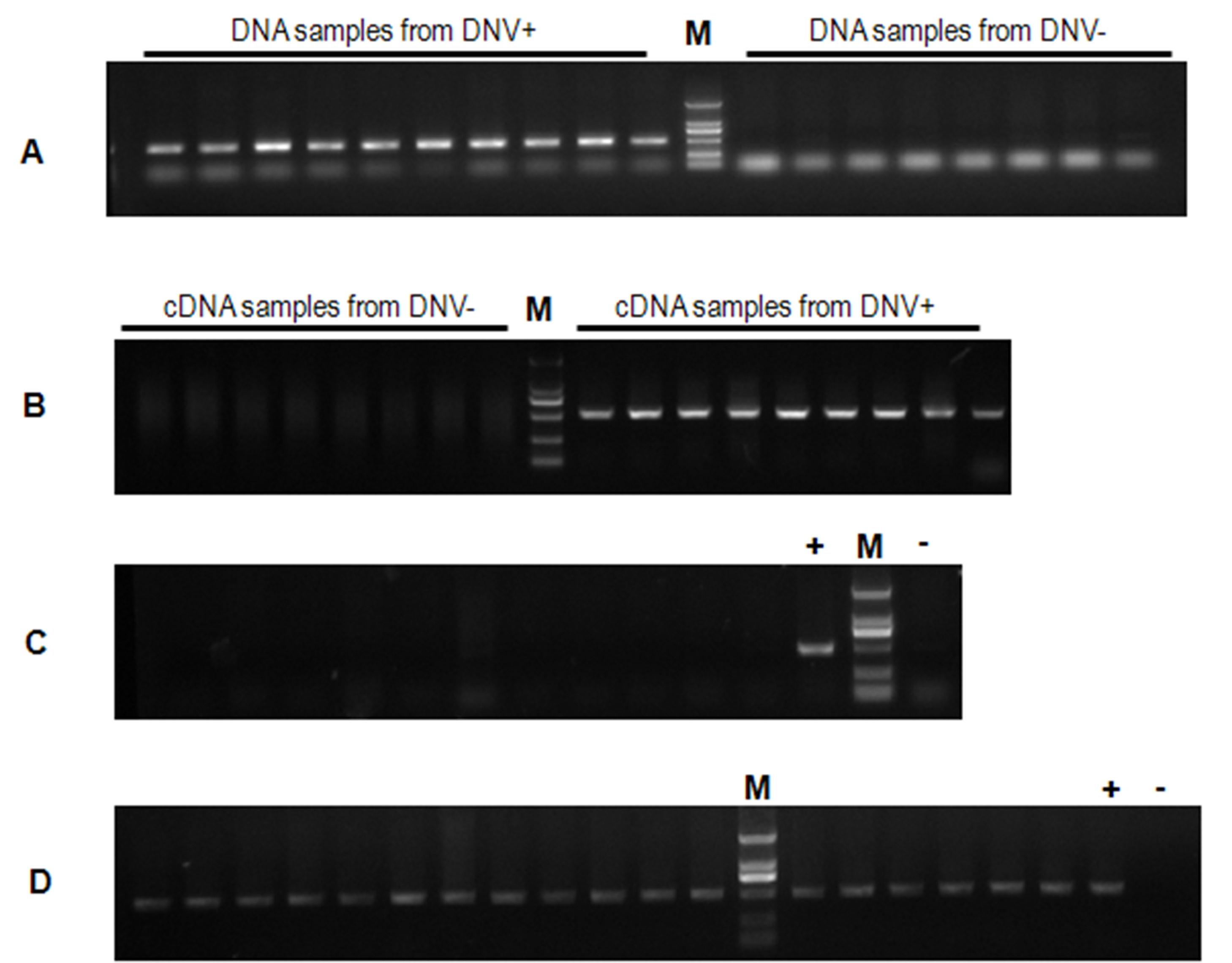

Supplement: Figure S3 — The detection of HaDNV-1 in sample used in bioassay. The detection of HaDNV-1 both (a) DNA and (b) RNA in 5th instar larvae infected by HaDNV-1 via oral inoculation in the bioassay experiment. The detection of HaDNV-1 in adults from (c) NONINF-strain and (d) INF-strain used in the experiment of egg production. M = marker (Figure 1b), “+” = positive control, “−” = negative control. (TIF) [file ppat.1004490.s003.tif]

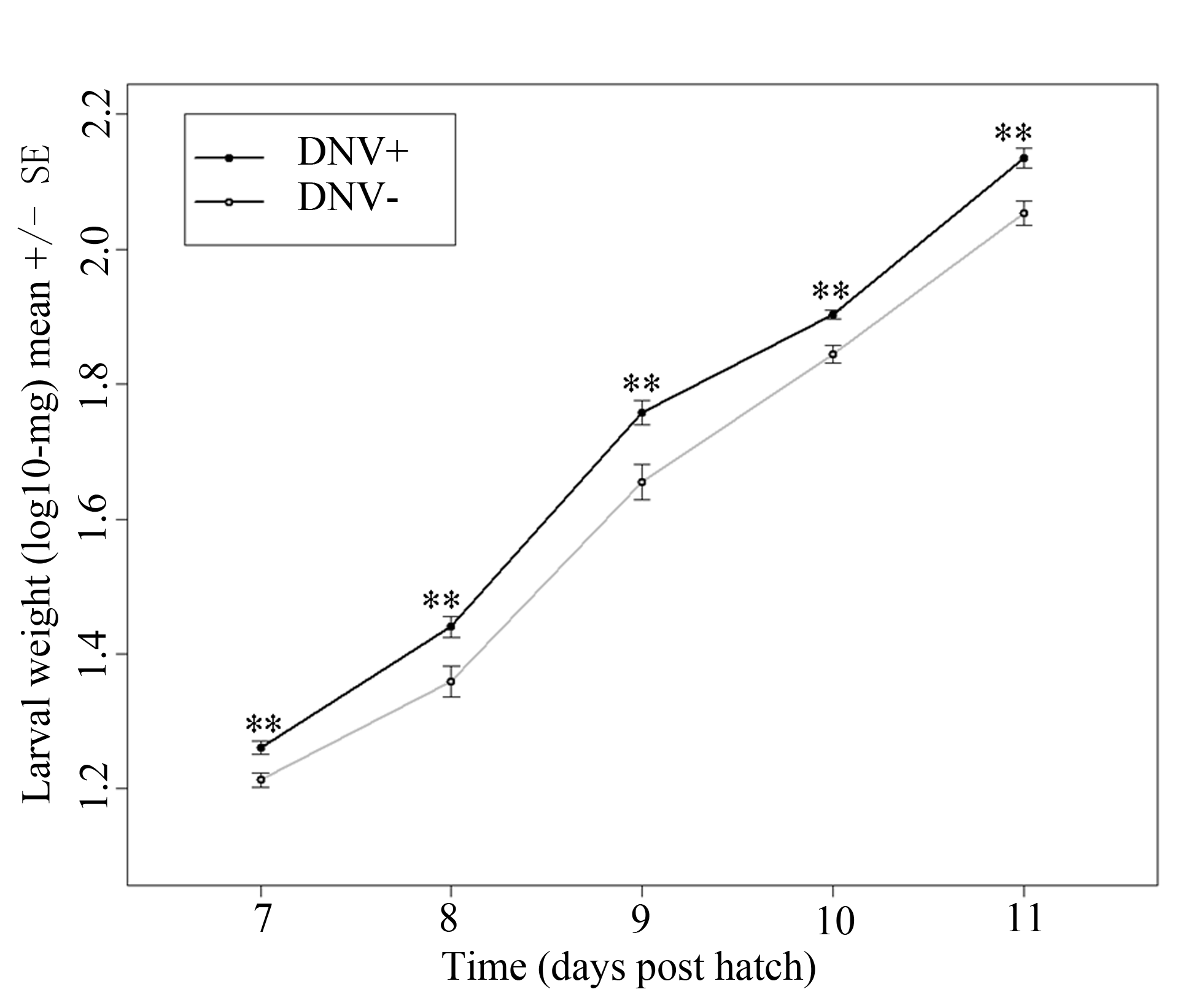

Supplement: Figure S4 — Larval weight (log10-transformed) from day 7 to 11 after hatching. For day 7, n = 7; day 8, n = 19; day 9, n = 19; day 10, n = 19; day 11, n = 19. DNV− = densovirus negative larvae, DNV+ = densovirus positive larvae. Means ± SE. * = P<0.05, ** = P<0.01. (TIF) [file ppat.1004490.s004.tif]

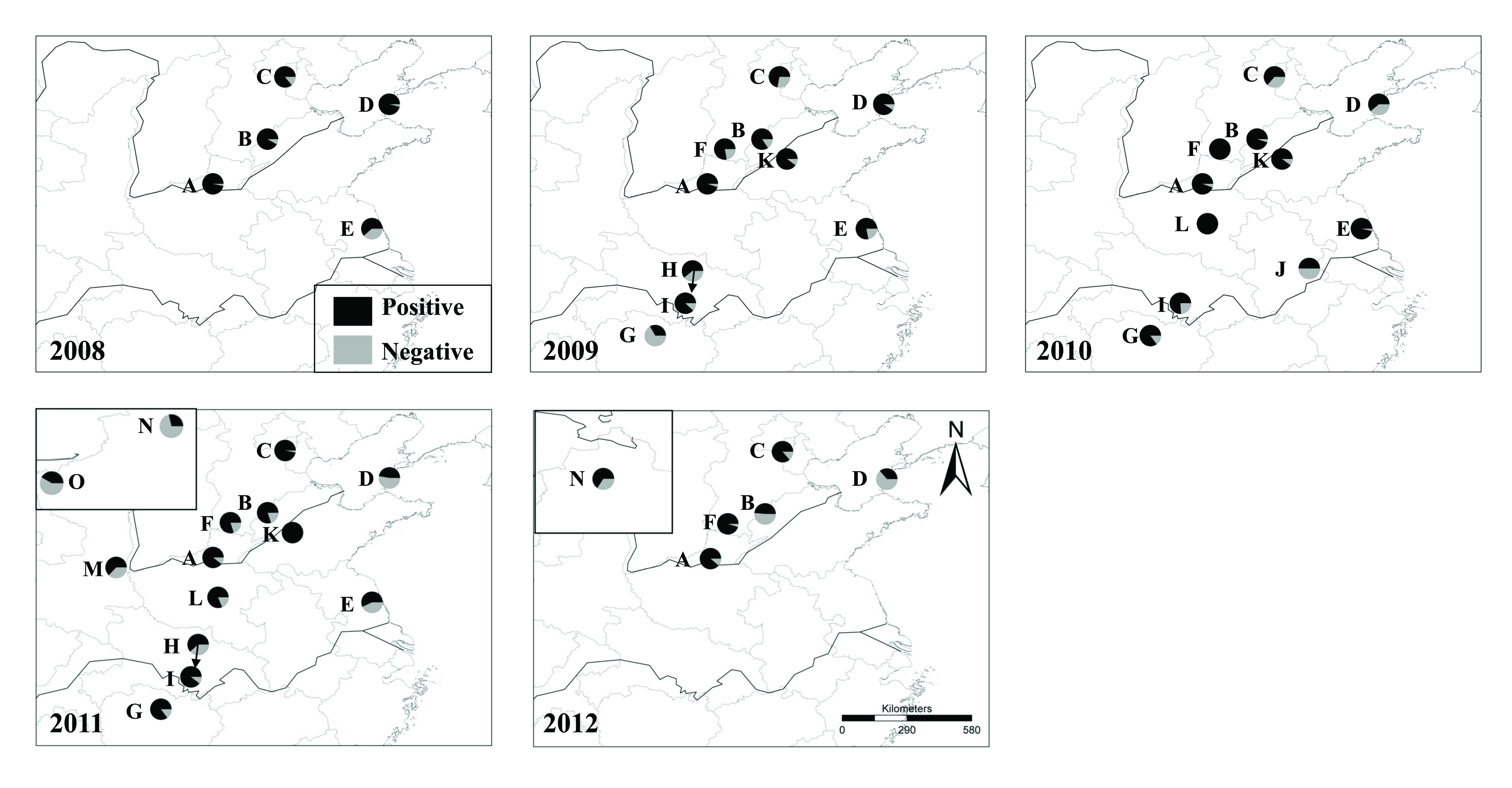

Supplement: Figure S5 — Distribution of HaDNV-1 in H. armigera from different populations. The black proportion of circles stands for infected individuals, and the gray stands for uninfected individuals. Different letters stand for different places for collecting samples. Infection rates were 87.1% in 2008 (n = 170), 81.2% in 2009 (n = 373), 76.8% in 2010 (n = 699), 68% in 2011 (n = 544) and 67% in 2012 (n = 370). Infected size/uninfected size in 2008: A = 30/1, B = 33/3, C = 43/7, D = 26/1, E = 16/10; in 2009: A = 38/2, B = 27/5, C = 18/7, D = 113/11, E = 39/11, F = 14/4, G = 8/16, H = 14/9, I = 15/2, K = 18/2; in 2010: A = 101/6, B = 98/5, C = 70/40, D = 113/72, E = 26/1, F = 18/0, G = 17/3, I = 22/7, J = 25/25, K = 27/3, L = 20/0; in 2011: A = 17/2, B = 8/2, C = 95/3, D = 46/51, E = 12/9, F = 16/4, G = 23/4, H = 8/5, I = 17/2, K = 18/0, L = 13/3, M = 63/37, N = 4/10, O = 30/42; in 2012: A = 16/2, B = 37/39, C = 127/20, D = 28/49, F = 19/1, N = 21/11. (TIF) [file ppat.1004490.s005.tif]

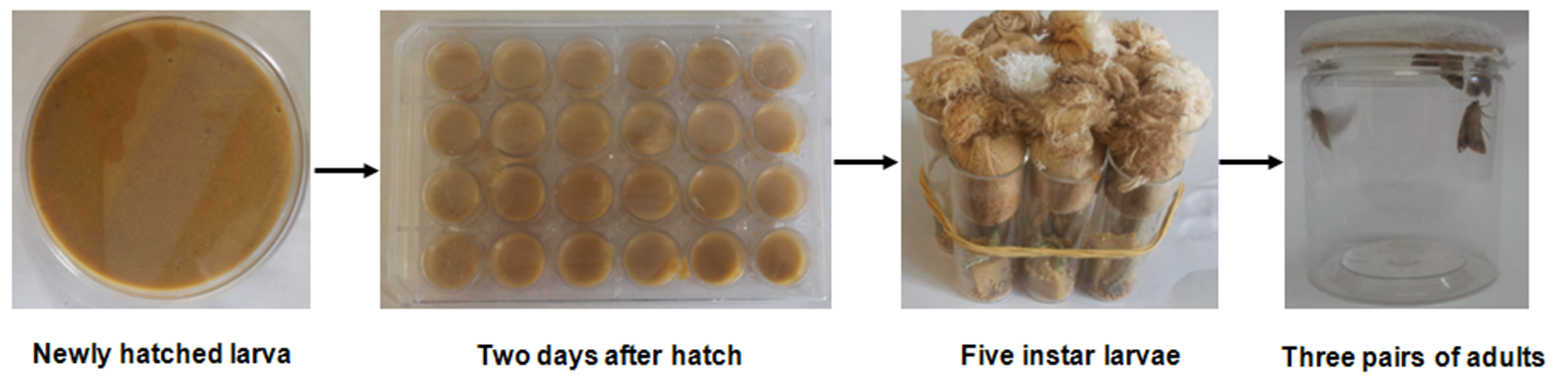

Supplement: Figure S6 — Tools used in the bioassay. (TIF) [file ppat.1004490.s006.tif]
