# Supplementary material for: Densovirus Is a Mutualistic Symbiont of a Global Crop Pest (Helicoverpa armigera) and Protects against a Baculovirus and Bt Biopesticide
Source: PLoS Pathog. 2014 Oct 30;10(10):e1004490. doi: 10.1371/journal.ppat.1004490 (PMC4214819; doi:10.1371/journal.ppat.1004490)
Supplement: Table S1 — The detection of DNV and NPV in larvae of H. armigera . “+” = infected, “−” = non-infected. “DNV+ and NPV+” = insects co-infected with both viruses. (DOC) [file ppat.1004490.s007.doc]

**Table S1** The detection of DNV and NPV in larvae of *H.armigera*. "+"=infected, "-"=non-infected. "DNV+ and NPV+"=insects co-infected with both viruses

| Place | Year | DNV+ | DNV- | NPV+ | NPV- | DNV+ and NPV+ |
| --- | --- | --- | --- | --- | --- | --- |
| Jinan | 2012 | 55 | 1 | 9 | 47 | 9 |
| Dezhou | 2012 | 6 | 54 | 33 | 27 | 4 |
| Taian | 2012 | 49 | 23 | 46 | 26 | 33 |
| Cangzhou | 2012 | 10 | 49 | 52 | 7 | 9 |
| Maanshan | 2012 | 26 | 32 | 27 | 31 | 11 |
| Tianmen | 2012 | 29 | 26 | 31 | 24 | 17 |
| Qianjiang | 2012 | 2 | 58 | 57 | 3 | 2 |
| Luohe | 2013 | 41 | 13 | 2 | 52 | 2 |
| Luoyang | 2013 | 22 | 24 | 0 | 46 | 0 |
| Langfang | 2013 | 15 | 9 | 1 | 23 | 0 |
| Nanyang | 2013 | 9 | 15 | 2 | 22 | 1 |
| Cangzhou | 2013 | 9 | 15 | 3 | 21 | 0 |
| Yuanyang | 2013 | 20 | 4 | 0 | 24 | 0 |
