# Supplementary material for: Densovirus Is a Mutualistic Symbiont of a Global Crop Pest (Helicoverpa armigera) and Protects against a Baculovirus and Bt Biopesticide
Source: PLoS Pathog. 2014 Oct 30;10(10):e1004490. doi: 10.1371/journal.ppat.1004490 (PMC4214819; doi:10.1371/journal.ppat.1004490)
Supplement: Table S2 — Primers used in this study. (DOC) [file ppat.1004490.s008.doc]

**Table S2** Primers used in this study.

| Primer name | Primer sequence (5'-3') | Location (nt) | Instruction |
| --- | --- | --- | --- |
| DVVPF | GGATTGGCCTGGGAAATGAC | 3703-3722 | The detection of HaDNV-1 |
| DVVPR | CGTTGTTTTTATATCCGAGG | 4179-4198 |
| PF | GGACAATGCTGGTGAGGC | 2970-2987 | Constructing the plasmid for standard curve |
| PR | AATCCTCTTTGTCCGTTATCTATG | 4110-4133 |
| VPF | CTGGTGAGGCGATGGACATG | 2978-2997 | Quantification of HaDNV-1 |
| VPR | TGACAAGATCCAGCGTAGACATC | 3073-3095 |
| VP-probe | (FAM) CCACCACTAGAGGACCCACCAGATG (Eclipse) | 3029-3053 |
| NPVF | AAAAGAAAAGCCAGCAACG | 573-591 (ORF14) | The detection of HaNPV and constructing the plasmid for standard curve |
| NPVR | ACGATTGAATGTCACCACG | 999-1017 (ORF14) |
| NPVF-p | AACAAGTACATCTAAAACCGCAACAT | 686-711(ORF14) | Quantification of HaNPV |
| NPVR-p | TGTACGAGCGGCATTACATTT | 777-797(ORF14) |
| NPV-probe | (FAM)CGCAGCCACGAGACCCGTCC (Eclipse) | 713-732(ORF14) |
